# Supplementary material for: Thienopyrimidine amide analogs target MmpL3 in Mycobacterium tuberculosis
Source: Antimicrob Agents Chemother. 2025 Sep 22;69(11):e00980-25. doi: 10.1128/aac.00980-25 (PMC12587563; doi:10.1128/aac.00980-25)
Supplement: Supplemental material — Fig. S1 and S2. [file aac.00980-25-s0001.docx]

**Figure S1.**

**Figure S2.**

**Figure S1.** **Exposure to TPA analogs induces cell wall stress in *M. tuberculosis*.**

*M. tuberculosis* P_iniBAC-_Lux was exposed to compounds for 72h and luminescence was read. Data are representative of two independent experiments (see Fig 2).

**Figure S2. TPA analogs boost ATP in *M. tuberculosis*.**

*M. tuberculosis* was exposed to compounds for 24h and ATP was measured using BacTiter-Glo. Growth was measured by OD after 5 d. Data are representative of two independent experiments (see Fig 3). Q203 was used as a control. Data were normalized to positive and negative controls
